# Mumps Vaccine Effectiveness Against Orchitis

**DOI:** 10.3201/eid1801.111178

**Published:** 2012-01

**Authors:** Susan Hahné, Jane Whelan, Rob van Binnendijk, Corien Swaan, Ewout Fanoy, Hein Boot, Hester de Melker

**Affiliations:** National Institute for Public Health and the Environment, Bilthoven, the Netherlands

**Keywords:** Mumps, mumps vaccine, MMR vaccine, orchitis

**To the Editor:** Yung et al. reported in the April 2011 issue of Emerging Infectious Diseases on the epidemiologic characteristics of the nationwide mumps outbreak in England and Wales in 2004−2005 (*1*). The associated effect of disease was considerable, with >43,000 reported cases and >2,600 hospitalizations. Compared with the prevaccine era, the average age of infection was higher, with infection occurring mostly in older teenagers and young adults (*2*). Older age at infection is associated with a higher risk of certain complications, particularly orchitis (*3*). Yung et al. reported that among cases of mumps, previous mumps measles rubella (MMR) vaccination offered considerable protection against orchitis, meningitis, and hospitalization (*1*).

In the Netherlands, mumps vaccination, using a 2-dose schedule with the MMR vaccine against measles, mumps, and rubella, was introduced in 1987, including catch-up vaccination of 3 birth cohorts (1983–1985). From birth cohort 1985 onwards, the coverage of the first and second dose of MMR has been consistently >92% (*4*). This coverage led to immediate control of mumps, with mumps related hospitalization dropping from 390 cases in 1987 to 11 in 1990 (*5*).

However, a major reemergence of mumps in the Netherlands occurred during August 2007–May 2009, when a large genotype D mumps outbreak affected mainly unvaccinated persons with a religious objection to vaccination (*6*). Subsequently, a genotype G outbreak of mumps started at the end of 2009, affecting mainly vaccinated adolescents. The outbreak started among university students in different cities, with a sudden increase in transmission after a large party for students in early 2009 (*7*).

The Dutch Centre for Infectious Disease Control advised Municipal Health Services in January 2011 to recommend MMR vaccination for university students who were unvaccinated or who had received only 1 dose of vaccine in the past. This policy was further implemented in the new academic year that began in August 2011. Information regarding the effectiveness of previous MMR vaccination against mumps complications is needed to support this policy and to predict the effect on mumps-related disease.

To study this policy, we analyzed mumps notifications in the Netherlands during December 1, 2009–June 14, 2011. Notifications include information about vaccination status and complications (e.g., orchitis, meningitis, encephalitis, pancreatitis). Vaccination status was confirmed by checking the national vaccination register, the general practitioner or patients’ vaccination booklets. Vaccine effectiveness against complications and hospitalizations was estimated by using logistic regression, adjusting for age group and sex.

In the study period, 958 cases were reported, and 16 case-patients were hospitalized (1.9% of case-patients with a known hospitalization status; n = 842). The median age of case-patients was 22 years (range 1–86 years), and 58.7% were male. We had information on the vaccination status of 905 case-patients (94.5%). For this group, 68% of these vaccination statuses were confirmed. Of the 905 case-patients, 16% were unvaccinated, and 10% and 68% had received 1 and 2 doses, respectively; 6% were vaccinated at least once, but number of doses was unknown. Of case-patients with information on the occurrence of complications (95.7%, n = 917), 73 (8.0%) reported >1 complication. Orchitis was by far the most frequently reported complication (66 case-patients, 11.8% of men). Other complications included pancreatitis (2, 0.2%), meningitis (3, 0.3%), and thyroiditis (1, 0.1%).

Previous vaccination with 1 or 2 doses reduced the risk for mumps orchitis among male mumps case-patients >12 years of age by ≈70% (Table). This finding is consistent with that reported by Yung et al. (*1*). Because of a lower number of cases, we could not reliably estimate the effect of vaccination in preventing hospitalization and other complications. The estimated proportion of case-patients hospitalized derived from the enhanced mumps surveillance by Yung et al. is remarkably similar to our estimate (3% and 2%, respectively). It is likely that we underestimated the overall effect of disease associated with this outbreak. Notification is known to be incomplete and complications developing after the date of notification are not included. However, because the reporting of complications is unlikely to be associated with vaccination status, we believe our estimates of the vaccine’s protective effects among cases of mumps are unbiased.

**Table:** Mumps complications by MMR vaccination status, the Netherlands, December 1, 2009–June 14, 2011*

Complication	MMR doses received	No. mumps cases	No. (%) cases with complications	OR	aOR†	p value	Adjusted VE,‡ % (95% CI)
Orchitis§	0	86	20 (23)	Ref	Ref	Ref	Ref
	1	48	5 (10)	0.38	0.34	0.05	66 (1 to 88)
	2	338	31 (9)	0.32	0.26	<0.01	74 (49 to 87)
Other complications¶	0	117	1 (1)	Ref	Ref	Ref	Ref
	1	85	1 (1)	1.38	0.88	0.93	12 (–14 to 95)
	2	571	6 (1)	1.23	0.75	0.80	25 (–5 to 91)
Hospitalization	0	130	4 (3)	Ref	Ref	Ref	Ref
	1	83	2 (2)	0.80	0.70	0.69	30 (–312 to 88)
	2	535	6 (1)	0.40	0.43	0.25	57 (–84 to 90)

*Only those for whom complication and vaccination status were known are included; therefore, totals may differ. MMR, mumps, measles, rubella; OR, odds ratio; aOR, adjusted odds ratio; VE, vaccine effectiveness; ref, reference categories. †OR and VE adjusted for age group (<18, 18−25, >25 y) and sex, except for orchitis, where the OR and VE were adjusted only for age group. ‡VE = 1 – OR where the OR is an approximation of the relative risk. §Only men ≥12 years of age are included. ¶Includes the following reported complications: pancreatitis (n = 2), meningitis (3), thyroiditis (1), bronchitis (1), high fever and shortness of breath (1).

Whereas objection to vaccinate was the predominant cause for the 2007–2009 mumps outbreak in the Netherlands, the current outbreak seems to be caused by secondary vaccine failure. Potential causes of this failure include waning of vaccine induced immunity, a relative mismatch between vaccine and outbreak strain, and intense social contact in the affected group (*8*). Our observations that orchitis was the most frequently reported complication, and that previous MMR vaccination considerably reduced the risk of orchitis among cases of mumps, are important to justify recommending mumps vaccination to unvaccinated persons.
